# MDACP: A Pathogen Genome and Metagenome Analysis Cloud Platform

**DOI:** 10.3389/fgene.2020.01007

**Published:** 2020-08-31

**Authors:** Na Han, Jiaojiao Miao, Tingting Zhang, Yujun Qiang, Xianhui Peng, Xiuwen Li, Wen Zhang

**Affiliations:** ^1^State Key Laboratory for Infectious Disease Prevention and Control, Chinese Center for Disease Control and Prevention, National Institute for Communicable Disease Control and Prevention, Beijing, China; ^2^Collaborative Innovation Center for Diagnosis and Treatment of Infectious Diseases, Hangzhou, China

**Keywords:** microorganism, genome, pathogen, web resource, analysis cloud platform

## Abstract

Pathogenic microorganism analysis based on next-generation sequencing technology is an important tool for clinical diagnosis, public health surveillance, and outbreak investigation. However, scientific researchers without the relevant background lack the time, training, or infrastructure to use large data sets or install and use command line tools. Therefore, the bioinformatic team at the Chinese Center for Disease Control and Prevention developed the Microbial Data Analysis Cloud Platform (MDACP) as a safe, professional, and efficient pathogen genetic data analysis platform for rapid microbial data mining, such as for candidate pathogen detection, genome typing, and traceability. MDACP is a web service system based on the Docker platform and can be used for data analysis on various operating systems. The platform focuses on pathogen analysis and continuously develops new analysis processes according to the analysis needs of the users. This platform has a friendly user interface and is easy to operate, allowing users to submit data through data pages or graphical clients, flexibly control parameters according to data conditions, and analyze data in parallel with multiple tasks. Researchers can quickly carry out bioinformatic analyses without coding work, promote follow-up research and information mining of projects, and improve the utilization of big data in the field of disease control. MDACP enables research personnel to conduct data analysis and management and assists clinicians and disease control personnel with mining information, such as pathogen identification, classification, and traceability.

## Introduction

Infectious diseases seriously threaten human health and are an important consideration for ensuring national biosafety. Infectious diseases also impact animals and plants, which may have a major effect on animal husbandry and agriculture. Introduction of foreign pathogens can cause human infectious diseases, as well as animal and plant diseases and even ecological disasters. Accurate and rapid detection and identification of pathogens and their resistance phenotypes is key to preventing and controlling infectious diseases. Traditional pathogen detection and identification technologies are mainly based on the immune response or nucleic acid amplification and hybridization, which can only detect one or a few pathogens at a low cost and efficiency and with a long experimental cycle; moreover, very few types of pathogens can be routinely detected. With the rapid development of genomic technology, particularly genome sequencing technology, and the advent of the big data era, it has become possible to detect and identify pathogenic microorganisms (Deurenberg et al., 2017).

Next-generation sequencing (NGS) is a powerful tool in medical microbiology and provides operational information that is difficult or impossible to achieve with traditional microbial technology. This method is widely used in studies of clinical and public health (Motroa and Moran-Gilad, 2017; Besser et al., 2018; Rossen et al., 2018). However, the use of NGS platforms requires sequencers to generate high quality and reliable sequencing data, as well as the means to analyze and interpret the large data sets generated. Analysis of large data sets often requires a combination of bioinformatics skills and high computational resources, making this approach impractical for many diagnostic medical microbiology laboratories (Deurenberg et al., 2017). Additionally, researchers who are unfamiliar with bioinformatic sequence analysis experience difficulty in determining the most appropriate protocol for achieving their research aims, selecting and applying the best bioinformatic tools, and identifying IT resources to access, store, and process large amounts of related to sequence data (Agrawal et al., 2017).

To overcome these challenges, the bioinformatics team at the Chinese Center for Disease Control and Prevention (China CDC) developed the Microbial Data Analysis Cloud Platform (MDACP). MDACP is a secure, professional, and efficient pathogen genetic data analysis platform that performs rapid and professional microbial data mining, such as candidate pathogen detection, for disease system practitioners and clinicians at all levels. MDACP simplifies the transfer, analysis, and visualization of large microbial data sets, integration of users through data pages or graphical clients, and transfer of publicly available data sets into analytics workflows. It also allows users to adjust the parameters set by workflow developers according to their experience levels or data situation, and to use cloud-computing resources to analyze data simultaneously with multi-tasking. MDACP is a pathogenic microbial data analysis cloud platform that does not require bioinformatics, IT technology, and computing resources. China CDC personnel can quickly carry out bioinformatic analyses, promote follow-up research and information mining of projects, and improve the utilization of big data in the field of disease control.

## Platform Implementation

The MDACP system uses a browser/server mode to support a cross-platform and horizontal expansion. The web server uses Nginx to handle access control. The platform database is MongoDB, which uses the Flask development framework to handle service access. The system is hosted on a Linux server. The web interface was developed using Python and the graphical client was developed in Java. Tools and workflows were installed with Docker technologies. Currently, MDACP platforms deployed on Alibaba have no restrictions on the number of users when the elastic expansion framework is used for the computing node. The results also show that the system supports 100 users simultaneously running workflows.

## Data Transmission and Management

MDACP provides two methods for transferring data. Users can upload the data to be analyzed and download the result files individually through the web data page. Alternatively, we provide graphical clients that support Windows and Mac systems to facilitate bulk data transfer between local and cloud storage services. To shorten the data transmission time, the graphical client provides dedicated compression tools and breakpoint retransmissions for large files of raw sequencing data to support data transmission when the network is unstable. We also use data validation during bulk data transfer to ensure the accuracy and security of the user data. Testing results showed that the time required for transmission of 1 Gb data is ~10 min. The transmission speed in different regions also depends on the local network speed.

## User Rights Management

To protect the safety of personal data, the user must register and log in before using MDACP. The platform data management adopts an access permission isolation mechanism, and the user can only access the data of other users by receiving authorization, thereby further improving the data security for the user. User personal data are safely protected in MDACP by multiple real-time copies, which are retained even if the physical hardware corrupts the data. The system administrator has no access to the user’s data but can generate a new workflow in the MDACP platform to which specific users are granted access.

## Online Development for Analysis Workflow

**FIGURE 1:**
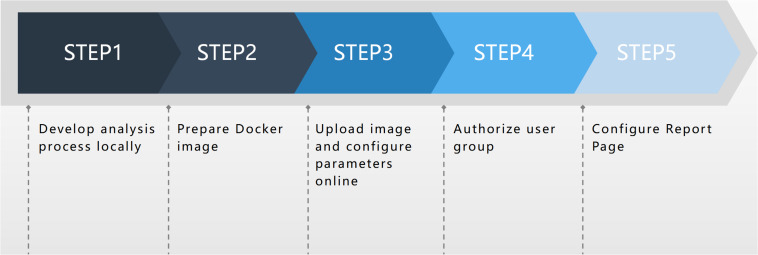
The online deployment process.

In contrast to the Seven Bridges and BaseSpace analysis platforms, MDACP analysis has flexible scalability and its analysis function module includes online configuration. Users with privileges can access the process configuration page of the platform and upload new workflows through simple procedures, parameter configuration, and other operations, as shown in Figure 1. The report page of the analysis process can also be customized according to the needs of the user.

MDACP is a web-based cloud platform that allows anyone with access to the Internet to perform pathogenic microbiological analyses without the need for local computing resources and expertise. In addition, MDACP uses Docker technology for analysis process encapsulation, which enables flexible expansion of computing resources, high availability, and good isolation, thereby improving the repeatability and practicability of the analysis process. MDACP has a user-friendly graphical interface and provides convenient pathogenic microbial analysis for inexperienced users, and standardized but configurable options maintain high functionality for experienced researchers.

Data are always saved in MDACP as multiple real-time copies, even if the physical hardware corrupts the data. In addition, the platform data management adopts an access permission isolation mechanism, and the user can only access the data of other users by receiving authorization, further improving data security.

## Analysis of Workflow and Application

MDACP is a computing environment based on Docker technology that configures complex software tools and workflows, allowing microbiologists or physicians with little experience in programming to quickly perform pathogenic microbiological data analyses in a web interaction mode. Compared with other research methods, and while generating analytical results without deviation, real-time and rapid analyses are important targets for pathogenic microbial data analysis. The development and published analyses of the bioinformatics team are the main deployment targets of the MDACP workflow. MDACP allows collaborative researchers to share their stable and biologically meaningful analysis tools for visual deployment with more users. In addition, the personalized analysis process, jointly developed according to the analysis needs of the user and open source software recommended by most users, is an important part of the MDACP workflow. In the future, MDACP will support user customization and workflow function sharing, and more analysis workflows will be open to users.

Currently, MDACP has opened 35 workflows, allowing disease control personnel and clinicians to perform pathogen identification and screen for drug resistance genes, fractals, and traceability. It also has analysis tools for raw read preprocessing, sequence assembly, gene prediction and annotation, specific analysis, and graphical transformation of results. We provide detailed functional descriptions, usage instructions, and referenced open source software for each workflow to help users quickly apply MDACP to perform data analysis and cite results.

According to the MDACP workflow, deployment conditions can be divided into the following three parts: (1) workflow developed and published by ourselves, including the 16SPIP workflow of pathogen identification for metagenomic samples (Miao et al., 2017), predictive process Effector search for secretory system effector proteins of pathogen genome type iii (Zhang and Bergelson, 2012), core genotyping of *Streptococcus suis* (Chen et al., 2013), and process of microbial genome evolution analysis based on genetic similarity ANItools (Zhang et al., 2014) and (2) cooperative researchers share the co-deployed analytical workflow, such as the metagenomic resistance gene detection process ARGs-OAP v2.0 (Yin et al., 2018). We then simplified the analysis steps of the process and supported simultaneous analysis of multiple samples to facilitate the determination of difference in resistance genes between samples; (3) several widely used bioinformatics tools, such as Prokka (Seemann, 2014) and Centrifuge (Kim et al., 2016). The MDACP platform connects these existing microbial genomic analysis tools into workflows on a point-and-click interface, which will be easier for user to use.

Previously, users needed Unix command line or high computing resources to run this bioinformatics software. Through our platform, users without programming experience or with limited computing resources can perform various steps of pathogenic microbial data analysis with various types of online MDACP workflows (Table 1) (Kent, 2002; Segata et al., 2011; Luo et al., 2012; Page et al., 2015; Wick et al., 2017).

**TABLE 1:** MDACP workflow.

**Categories**	**Workflow name**	**Scope of application**	**Function description**	**Related software in workflow**	**Time**
Pathogen detection	Guthealthy	16S metagenome raw data	This process can be used to evaluate the diversity of bacterial community (genus number, Shannon index, etc.) and the abundance of core bacteria in fecal samples, and identify the species of pathogenic bacteria that may be contained in the samples.	Bowtie2 (Langmead and Salzberg, 2012); Bwa (Li, 2013)	∼7 min^*a*^
	Histoplasm_cap sulatum_ detection	WGS metagenome raw data	Based on the metagenome sequencing results of clinical samples (fastq format), this workflow could identified whether there was Histoplasma capsulatum in the samples, and extracted the relevant sequences	Bowtie2 (BR11)	∼2 h^*b*^
	easyCentrifuge	WGS metagenome raw data	Centrifuge simplified online version of metagenome sequence classification software (only applicable to human and virus, not to bacteria and parasites)	Centrifuge (Kim et al., 2016)	∼10 min^*b*^
Data preprocessing	QCfilter	Fastq format	This process provides the screening of low-quality sequencing data. Through this process, users can screen out all the low quality sites in the original sequencing data and the data after screening, and get the quality control report after screening. Users can control the strictness of screening by setting parameters	Fastqc (Andrews, 2013)	∼5 min^*b*^
	Cat_sequences	Fasta format	Combining several FASTA sequences into one sequence	Perl	∼5 min^*c*^
Genome assemble	Mix_assemble	Fastq format	The workflow is used for the hybrid assembly of sequencing results from Illumina and nanopore, and can be used for the assembly of bacterial genome and plasmid.	Spades (Nurk et al., 2013)	∼2 h^*b*^
	Assemble_ Bacterial_ Genome_ stat	Fastq format	A one-stop analysis process for Bacterial genome assemble and evaluation. Based on Fastq sequencing files, the assembly and evaluation of bacterial genome were completed, and gene prediction and functional annotation were carried out based on the assembly results. Please view the analysis results on the download page.	Spades (Nurk et al., 2013); Quast (Mikheenko et al., 2018); Prokka (Seemann, 2014)	∼2 h^*b*^
Gene prediction and annotation	Glimmer	Genome (Fasta format)	Searching for gene regions in microbial genome sequences	Glimmer (Delcher et al., 2007)	∼10 min^*c*^
	Prokka	Genome (Fasta format)	A rapid tool for gene annotation of bacterial genome	Prokka (Seemann, 2014)	∼10 min^*c*^
Genome comparison	ANItools	Genome (Fasta format)	This process supports the rapid alignment of microbial genome sequences, and realizes the identification of the genetic similarity (ANI) of multiple microbial genome sequences of the same species in the database by one genome sequence of user self-sequencing within 10 min, and constructs the evolutionary relationship tree based on the ANI value	ANItools (Zhang et al., 2014)	∼30 min^*c*^
	Call_SNP	Genome (Fasta format)	This work flow could compare more than two bacterial genome sequences (up to 30 sequences) to find the core genes in all strains and identify SNP sites	Blast (Altschul et al., 1990)	10 min∼2 h^*c*^
Graphical display	Heatmap	Data matrix	Draw heatmap	R	∼5 min^*d*^
	Boxplot	Data matrix	Draw boxplot: Comparison of data distribution between groups	R	∼5 min^*d*^

*^*a*^The test input file is a fastq format file with 50,000 reads. ^*b*^The test input file is a 2 Gb file (fastq format). ^*c*^The test input file is a 5 Mb file (fasta format). ^*d*^The test input file is a matrix table (100 rows ^∗^ 100 columns).*

## Use of the Platform

MDACP simplifies and accelerates the generation of microbiological data analysis results, and the user-friendly interface saves time. The user can upload sequencing data to the system through the web data page or graphical client. When the data are verified and available, the user can run the workflow on the web workflow page and submit the analysis task. This platform allows the user to submit multiple analysis tasks at the same time, analyze the data simultaneously, and view the analysis report on the web task report page. For example, users can assemble their bacterial genome using the “Assemble_Bacteria_Genome_Stat” tool in three steps: (1) upload the fastq format sequencing files to MDACP; (2) choose the “Assemble_Bacteria_Genome_Stat” tool and select the input files; and (3) click the “Run” button and download the assembled genome after ~10 min. The report files are shown on the automatically refreshed page. MDACP has a user-friendly interface allowing users to submit data through data pages or graphical clients, flexibly control parameters according to data conditions, and analyze data simultaneously with multiple tasks. To date, 417 China CDC users have submitted more than 4,600 analysis tasks on this platform, which will continue to increase.

In contrast to other web-based platforms, such as Seven Bridges and BaseSpace, MDACP focuses on the microbiology field and supports various types of free analysis workflows based on the needs of clinicians and for communicable disease control. Compared with the MicrobiomeAnalyst (Chong et al., 2020) and MetaCoMET^1^ platforms, which support online analysis of biome tables generated by metagenomic analysis, the MDACP platform supports workflows involving direct analysis of raw sequencing data and can easily be applied in resource-limited situations as well as in clinical laboratories and public health lab settings where bioinformatics expertise is lacking. With our MDACP platform, users can easily perform several types of bioinformatics analysis on a point-and-click interface in 5 min–2 h (Table 1). The run time depends on the workflow chosen by the user.

This MDACP platform can not only facilitate data analysis and management for scientific research, but also assist clinicians and disease control personnel with information mining such as pathogen identification, classification, and traceability. The platform is free and available for research users at https://analysis.mypathogen.org.

## Data Availability Statement

Publicly available datasets were analyzed in this study. This data can be found here: https://analysis.mypathogen.org.

## Author Contributions

WZ designed this study and wrote this manuscript. WZ, NH, JM, XP, TZ, YQ, and XL contributed to data analysis and platform establishment. All authors reviewed the manuscript.

## Conflict of Interest

The authors declare that the research was conducted in the absence of any commercial or financial relationships that could be construed as a potential conflict of interest.

## Footnotes

1 https://probes.pw.usda.gov/MetaCoMET/

## Abbreviations

China CDC: Chinese Center for Disease Control and Prevention
MDACP: Microbial Data Analysis Cloud Platform
NGS: Next-generation sequencing.
